# Degree of Impurity and Carbon Contents in the Grain Size of Mg-Al Magnesium Alloys

**DOI:** 10.3390/ma16083069

**Published:** 2023-04-13

**Authors:** Sung-Su Jung, Yong-Ho Park, Young-Cheol Lee

**Affiliations:** 1 Energy Component & Material R&BD Group, Korea Institute of Industrial Technology, Busan 46938, Republic of Korea; 2 Department of Materials Science and Engineering, Pusan National University, Busan 46241, Republic of Korea

**Keywords:** AZ91 alloys, high-purity, grain refinement, carbon source, Al-C particle

## Abstract

In this study, the tendency of having different grain structures depending on the impurity levels in AZ91 alloys was investigated. Two types of AZ91 alloys were analyzed: commercial-purity AZ91 and high-purity AZ91. The average grain size of the commercial-purity AZ91 alloy and high-purity AZ91 is 320 µm and 90 µm, respectively. Thermal analysis revealed negligible undercooling in the high-purity AZ91 alloy, while undercooling of 1.3 °C was observed in the commercial-purity AZ91 alloy. A CS analyzer was employed to precisely analyze the carbon composition of both alloys. The carbon content of the high-purity AZ91 alloy was found to be 197 ppm, while the commercial-purity AZ91 alloy contained 104 ppm, indicating a difference of approximately 2 times. The higher carbon content in the high-purity AZ91 alloy is believed to be due to the use of high-purity pure Mg in its production (the carbon content of high-purity pure Mg is 251 ppm). To simulate the vacuum distillation process commonly used in the production of high-purity Mg ingots, experiments were conducted to investigate the reaction of carbon with oxygen to produce CO and CO_2_. XPS analysis and simulation results for activities confirmed the formation of CO and CO_2_ during the vacuum distillation process. It could be speculated that the carbon sources in the high-purity Mg ingot provide Al-C particles, which act as nucleants for Mg grains in the high-purity AZ91 alloy. Thus, it can be considered the main reason that high-purity AZ91 alloys have a finer grain structure than that of commercial-purity AZ91 alloys.

## 1. Introduction

Magnesium alloys have been attracting significant attention due to their advantageous properties such as low density, good castability, and specific strength. Among various magnesium alloys, aluminum-containing magnesium alloys, such as AM and AZ series, are mainly used in the industry due to their good castability and outstanding mechanical properties compared with other magnesium alloys [1,2]. However, due to their low tensile/yield strength and ductility compared with other competitive alloys such as aluminum alloys, it is essential to improve their mechanical properties [3]. 

Grain refinement is a typical approach for improving the mechanical properties of metallic materials. Moreover, it is an attractive approach for the simultaneous improvement of strength and ductility [4]. A representative grain refinement mechanism of magnesium alloys is the addition of zirconium [5]. However, since zirconium reacts with aluminum to form stable intermetallic compounds, the grain refinement effect of adding zirconium disappears in aluminum-bearing magnesium alloys. Thus, various studies have been conducted to find effective grain refinement methods for aluminum-bearing magnesium alloys. Several methods, such as the Elfinal process, carbon inoculation, RE addition, and melt superheating, have been reported to achieve effective grain refinement for aluminum-containing magnesium alloys [6,7]. However, among these methods, a peculiar phenomenon that contradicts the general nucleation theory of solidification has been reported [8]. This phenomenon is that the high-purity Mg-Al alloys manufactured using high-purity magnesium ingots have a finer grain size than the commercial-purity Mg-Al alloys manufactured using pure magnesium ingots, which are commercially available in the industry fields. This phenomenon was first reported by Nelson [9,10,11,12], and it is contrary to the theories of constitutional undercooling and heterogeneous nucleation. Tamura et al. [9,10] and Cao et al. [11,12] also observed that the grain size of high-purity Mg-Al alloys is generally finer than that of commercial Mg-Al-based alloys. Moreover, they reported that Al-C or Al-C-O intermetallics can act as nucleants for α-Mg grains and poisoning of the nucelants occurred due to impurities, such as Mn and Fe in commercial purity Mg-Al alloys. Despite extensive studies, a clear explanation has not been given on the phenomena that high-purity Mg-Al alloys have a finer grain size than commercial-purity Mg-Al alloys. Although the formation of Al-C or Al-C-O intermetallic particles, which are known to be powerful nucleants, is closely related to the carbon composition of Mg-Al alloys, a precise analysis of the carbon content in Mg-Al alloys has not been reported. In addition, there is also a lack of investigations on how carbon sources forming Al-C or Al-C-O intermetallic particles are introduced into high-purity Mg-Al alloys, as carbon is not an additive element in Mg-Al alloys.

Therefore, this study was conducted to delve deeper into the fine grain structure and its causes observed in high-purity Mg-Al alloys. The carbon contents in high-purity and commercial-purity Mg-Al alloys were precisely analyzed and intensive investigations were conducted on how the carbon sources were introduced into the high-purity Mg-Al alloy. In addition, how these carbon sources form Al-C or Al-C-O particles and how these particles affect grain refinement were identified. We aimed to propose new possibilities for grain refinement on high-purity Mg-Al alloys that have not been reported in previous studies.

## 2. Materials and Experiments

### 2.1. Materials and Casting Process

Two different AZ91 alloy types were prepared for this study. The high-purity AZ91 alloys used in this study were manufactured from 99.99% pure magnesium, 99.99% pure aluminum, and 99.99% pure zinc. Commercially available AZ91 alloys were used for the analysis of commercial-purity AZ91 alloys. First, 1 kg of each alloy was melted in an Al_2_O_3_ alumina crucible using a resistance furnace. Melting was conducted at 670 °C under protective gas of 1.0% SF_6_ and 99.0% N_2_. The melts were poured into a cylindrical steel mold preheated to 250 °C. The chemical composition of the samples was analyzed by an optical emission spectrometer (Spectro MAXx, SPECTRO, Germany). Table 1 shows the chemical composition of the alloy samples used in this study.

### 2.2. Measurement of Grain Size

The metallographic specimens were polished and etched using an acetic-picral etchant for clear color contrast of grain structure. The microstructure was observed using an optical microscope (Leica MC 170, LEICA, USA). The measurement of average grain size was conducted according to ASTM E112-10 on the central region of a transverse section of each sample.

### 2.3. Thermal Analysis

Thermal analysis experiments were conducted using cylindrical graphite crucibles in order to measure the undercooling during the solidification of alloys. The graphite crucible was immersed in the melt until the temperature of the graphite crucible reached the melting temperature of the molten metal. The crucible filled with the molten metal was transferred to the ceramic board and a K-type thermocouple, calibrated by measuring the equilibrium melting temperature of high-purity (99.99%) pure aluminum, was immersed into the center of the melt to record the temperature during the solidification process. The cooling curves were recorded by a data logger (NI cDAQ-9174, NATIONAL INSTRUMENTS, USA) at the frequency of 20 Hz.

### 2.4. Manufacturing of Rapidly Solidified Ribbon Samples and Particle Analysis

Rapidly solidified samples were prepared by melting 2 g of each alloy sample in a ceramic tube. Samples were melted using an induction coil and then injected into a copper wheel rotating at the speed of 1500 rpm. The temperature of each casting condition was precisely controlled by attaching a thermocouple to the ceramic tube. The microstructures and particles were investigated using a scanning electron microscope (Merlin compact, ZEISS, Germany).

### 2.5. Analysis of Carbon Composition and Experiment on Carbon Sources Generation

**Figure 1:**
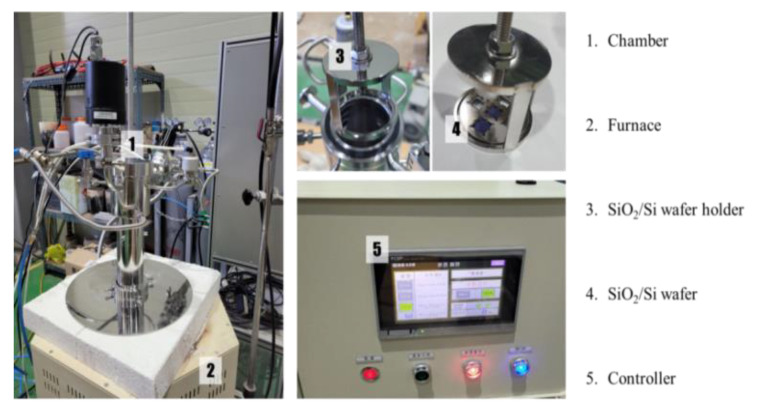
Apparatus for the experiment on carbon sources generation.

Figure 1 shows the schematic for the carbon distillation experiment apparatus for simulating the distillation process of Mg ingots. Milled carbon powders were filled at the bottom of the chamber and SiO_2_/Si wafers were installed on the top of the chamber. The chamber pressure was maintained at 10^−3^ torr and the bottom of the chamber was placed inside the furnace, which was maintained at 600 °C. A water jacket was installed on the top of the chamber in order to facilitate condensation of any gaseous materials. The surfaces of the SiO_2_/Si wafers were observed by AR XPS (Angle-Resolved X-ray Photoelectron Spectrometer, SPECTRO, Germany) to closely analyze any changes in the intensity of carbon picks before and after the experiment.

## 3. Results & Discussion

### 3.1. Grain Structure and Degree of Undercooling

**Figure 2:**
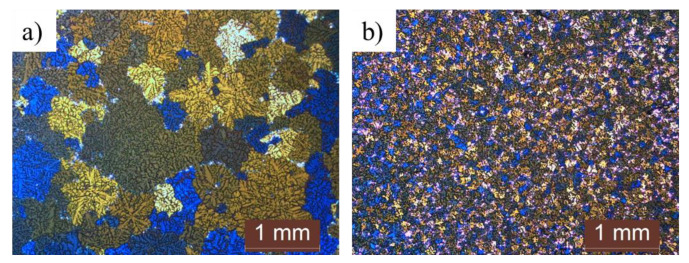
Microstructures of the as-cast samples: (**a**) Commercial-purity AZ91 alloy; (**b**) High-purity AZ91 alloy.

Figure 2 shows the microstructures of as-cast commercial-purity and high-purity AZ91 alloys. The average grain size of the commercial-purity AZ91 alloys and high-purity AZ91 alloys is 320 µm and 90 µm, respectively. Although both samples showed similar dendritic microstructures with equiaxed grains, the grain size of the high-purity AZ91 alloy is approximately four times finer than that of the commercial-purity AZ91 alloy. In addition, the microstructure of the high-purity AZ91 alloy is more uniform, with similar grains in size and shape. However, the microstructure of the commercial-purity AZ91 alloy showed high non-uniformity in terms of its grain shapes and sizes. The grain refinement effect caused by the constitutional undercooling of both alloys could be excluded because the compositions of Al and Zn, which are the main additives of both alloys, are similar. Additionally, the grain refinement effect due to the changes in the cooling rates can also be excluded, since both samples were manufactured under the same casting conditions. The composition analysis of both alloys shown in Table 1 indicates significant differences in the composition of Mn, Si, and Fe. Previous studies [13] have reported that the Si element forms a Mg_2_Si phase in AZ91 alloys, which affects the grain refinement of AZ91 alloys. However, the Si content of at least 0.2 wt.% is considered the minimum amount required to observe any distinguished grain refinement effect, thus the grain refinement effect of Si could be ignored in this study. Generally, Mn is added to AZ91 alloys to eliminate Fe, which deteriorates the corrosion resistance of the alloy. The studies of Cao [11] and Han [14] have reported that Al-Mn-(Fe) compounds formed by Mn addition affect the grain refining of AZ91 alloys. However, Mn and Fe elements were also reported to poison the nucleations in AZ91 alloys [9,15], and the effect of Mn and Fe on the grain refinement of AZ91 alloys is still a subject of debate among many researchers. This study was conducted with a focus on the nucleants of both alloys, which is different from previous studies that focused on the compositional differences between high-purity and commercial AZ91 alloys.

**Figure 3:**
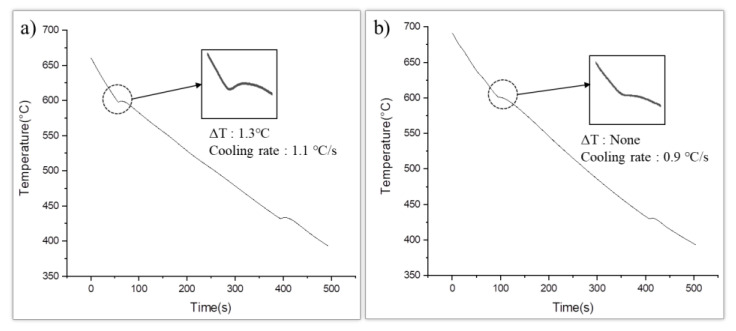
Cooling curves of: (**a**) the commercial-purity AZ91 and (**b**) high-purity AZ91 alloy.

Figure 3 shows the cooling curves of the commercial-purity and high-purity AZ91 alloys. The presence of nucleants in the melt could be determined by observing the undercooling degree of the alloys during solidification. When powerful nucleants existed in the melts, the thermodynamic driving force required to form a solid phase from the liquid phase was lowered so that the nuclei of the solid phase could be easily formed with a small degree of undercooling [7,16]. During solidification, an undercooling of 1.3 °C was observed in the commercial-purity AZ91 alloy, and a negligible undercooling was observed in the high-purity AZ91 alloy. Thus, it can be concluded that the high-purity AZ91 alloy had powerful nucleants that could be activated for nucleation with little activation energy. A quantitative analysis of the nucleants was not performed in this study. However, the undercooling degree of the alloys can be the basis for the qualitative interpretation that the high-purity AZ91 alloy had more nucleants that affected grain refinement than the commercial-purity AZ91 alloy. The cooling rate was a major factor influencing the undercooling degree, and the measured cooling rates of both samples were 1.1 °C and 0.9 °C, respectively. From this result, it was concluded that the effect of the cooling rates on the undercooling changes shown in this study is negligible [16].

### 3.2. Carbon Contents

Table 1 shows the chemical compositions of the commercial-purity and high-purity AZ91 alloys. The chemical composition results show that the aluminum and zinc compositions are within the specifications of the AZ91 alloy, as defined by ASTM. Although there is a slight difference in the compositions of the two elements, the changes in the constitutional undercooling degree produced by the two alloying elements can be neglected. The compositions of the Mn, Si, Fe, and Cu elements were found to be higher in the commercial-purity AZ91 alloy than in the high-purity AZ91 alloy. According to the general heterogeneous nucleation theory [8], impurity elements or the compounds formed by impurity elements more likely act as nucleants for α-Mg grains. However, as shown in Figure 2, the grain size of the high-purity AZ91 alloy with a low level of impurities is finer than that of the commercial-purity AZ91 alloy. The main reason for this phenomenon, which is contrary to the general nucleation theory, is the carbon content level in the samples. Previous studies [9,10,11,12] have suggested that Al-C or Al-C-O particles are the main cause of grain refinement in high-purity AZ91 alloys. Tamura et al. [9] suggested that the composition of carbon is ~20 ppm. However, they did not explain how they measured the carbon content level. It is very difficult to precisely analyze carbon contents using general analysis methods, such as OES and ICP. 

**Figure 4:**
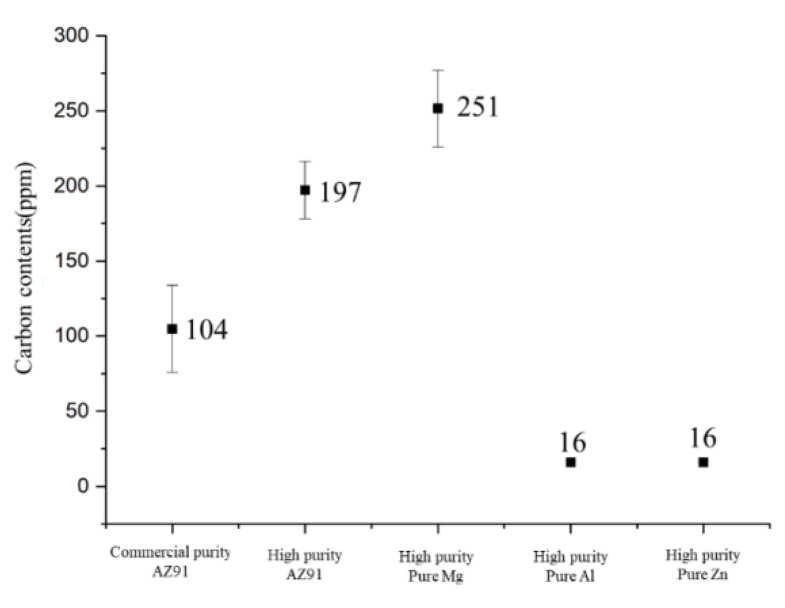
Carbon contents of commercial-purity AZ91, high-purity AZ91 and high-purity pure Mg ingot used for manufacturing high-purity AZ91 alloys.

Figure 4 shows the carbon contents of commercial-purity AZ91, high-purity AZ91 and high-purity pure Mg ingot analyzed by the CS analyzer. Generally, CS analyzer is used to analyze carbon level in ferrous materials. However, by optimizing the voltage and material weight of the CS analyzer, we managed to analyze the carbon levels in Mg alloys. The average carbon composition in the commercial-purity AZ91 alloy was 104 ppm and 197 ppm was detected in the high-purity AZ91 alloy. Several experiments were conducted repeatedly and the results clearly showed that the carbon composition in the high-purity AZ91 alloy was higher than that in the commercial-purity AZ91 alloy. In addition, the carbon composition of those high-purity pure Mg, high-purity pure Al, and high-purity pure Zn, which were used for manufacturing the high-purity AZ91 alloys, were detected to be 251 ppm, <16 ppm, and <16 ppm, respectively. From these results, it can be concluded that the high carbon level of the final high-purity AZ91 alloy was inherited from the high-purity pure Mg ingot. Through observations of the microstructure and degree of undercooling, it was predicted that high-purity AZ91 alloys might have nucleants with high potency for the nucleation of α-Mg grains, and it could be concluded that those nucleants were likely to contain carbon sources considering the precise analysis results of the carbon content in the alloys.

### 3.3. Particles in the Rapidly Solidified Microstructure

Considering the CS analysis of the alloys, it could be suggested that particles containing carbon would affect the grain structure of high-purity AZ91 alloys. Typically, precipitated intermetallic compounds and eutectic phases such as Mg_17_Al_12_ are observed in the microstructure of permanent mold castings due to the low cooling rates. This makes it very difficult to identify the origin of the particles or compounds that exist in the melt, and to determine their effects on the grain size changes of the alloys. Additionally, it is difficult to identify nucleants with a size of hundreds of nanometers existing in three-dimensionally grown dendritic-grains. In this study, we prepared thin-ribbon samples using a rapid solidification process to clearly identify compounds or particles that might act as nucleants in AZ91 alloys. The cooling rate of a rapidly solidified alloy ribbon was calculated to be 1 × 10^6^ °C/s [17]. This is a typical experimental process to prevent the diffusion of solute elements and segregation during the solidification process. Using the rapid cooling process, it is possible to obtain microstructures in which precipitates and eutectic phases are not fully developed and in which compounds and particles in the liquid phase can be clearly identified [10].

**Figure 5:**
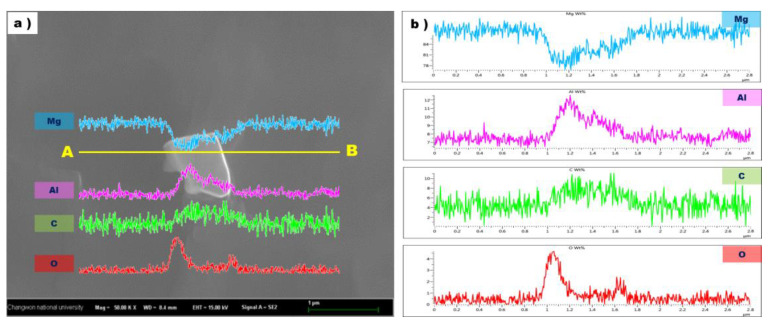
Particle observed in high purity AZ91 ribbon sample (**a**) and the EDS line profile for this particle (**b**).

Figure 5 shows one of the particles in a ribbon sample of a high-purity AZ91 alloy and the elemental distribution results along the A-B line across the particle. The EDS line scanning results confirmed that the particle was enriched in aluminum and carbon. The high magnesium pick can be explained by the particle size and resolution of the beam. The size of the particle was about 500 nm~1 µm, which is far smaller than the minimum range of EDS analysis resolution and the high magnesium pick would come from the matrix around the particle. However, the weight percent of magnesium in the particle was relatively small compared to the Mg matrix. In addition, it was thought that some part of the particle surface was oxidized during the manufacturing and pretreatment of the ribbon samples. This would explain the high level of oxygen detected at the edge of the particle. From these results, it could be deduced that Al-C particles were present in the Mg molts as a solid phase.

**Figure 6:**
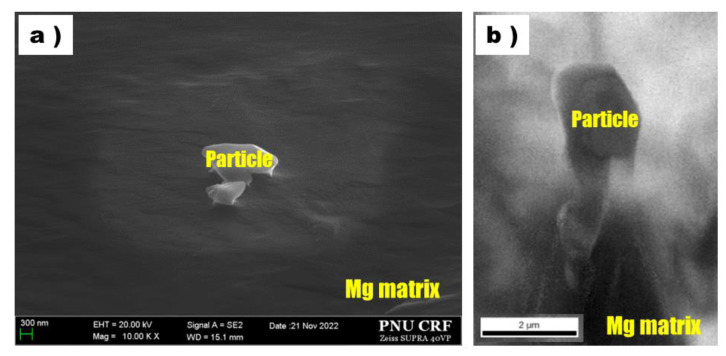
Particle on high purity AZ91 alloy ribbon samples observed using EBSD: (**a**) SEM image and (**b**) IQ map.

In order to identify the origin of particles more clearly, one of those Al-C particles was investigated by EBSD and the analysis results are shown in Figure 6 with an SEM image and IQ map. Since the particle observed in this EBSD analysis was protruded from the Mg matrix, clear grain orientation data were not obtained from EBSD phase mapping as this involved tilting the specimen at a high angle. However, based on the line scan results in this study, it could be suggested that Al-C particles could be present in the high purityAZ91 alloy melt and act as the effective nucleates for α-Mg grains. We intend to carry out a clear analysis of the crystal structure and composition of the Al-C particles observed in the rapidly solidified sample through additional research. In this study, we focused on the reason for the presence of Al-C particles in high-purity AZ91 alloy samples even though no carbon source or Al-C particles were added to the melt.

### 3.4. Carbon Sources

**Figure 7:**
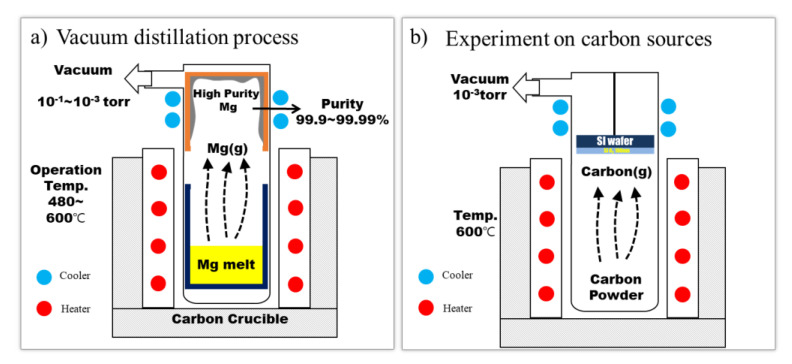
Schematic of the vacuum distillation process (**a**) and the experiment on carbon sources generation (**b**).

Generally, commercial-purity pure magnesium crowns are manufactured by the Pidgeon process through the thermal reduction of dolomite ore [18]. The purity of these pure magnesium crowns is between 99.5 and 99.7% and they are mainly used for the production of commercial-purity magnesium alloys [19,20]. These commercial-purity pure magnesium crowns are used in the manufacture of high-purity pure magnesium crowns through additional vacuum distillation [21,22,23]. Figure 7a shows the manufacturing process of high-purity pure magnesium ingots. It was found that carbon crucibles are generally used for manufacturing high-purity pure Mg ingots [21,24] and there is a possibility that carbon sources would be introduced into high-purity pure magnesium crowns. The vacuum distillation process, which is used to produce high-purity Mg crowns, involves magnesium vaporization for a considerably long time at 600 °C under a vacuum environment of 10^−3^ torr [24]. Although carbon is a very stable element, it is considered that some types of carbon sources can be produced from carbon crucibles through reactions with the elements in manufacturing environments. Figure 7b shows a schematic illustration of a carbon vaporization experiment device. Carbon powders were charged into the chamber, which was maintained at 600 °C in a vacuum of 10^−3^ torr. This condition is similar to the general vacuum distillation process used in the manufacture of high-purity pure Mg ingots [24]. The qualitative changes in the carbon on the surface of the SiO_2_/Si wafers with the carbon vaporization experiment were measured using an X-ray photoelectron spectrometer.

**Figure 8:**
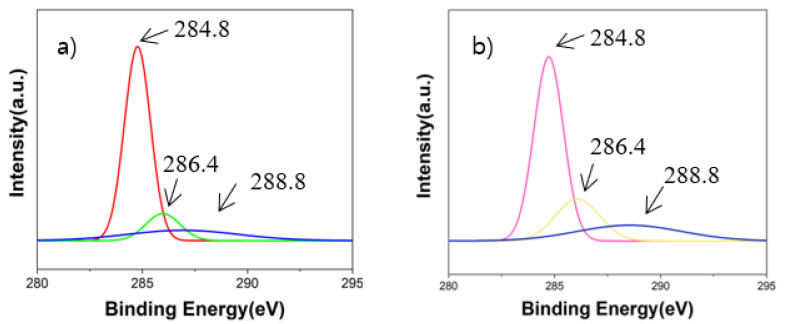
Binding energy peaks of the carbon atoms and carbon compounds on the SiO_2_ wafer: (**a**). Base; (**b**) After vacuum carbon vaporization experiment.

**Figure 9:**
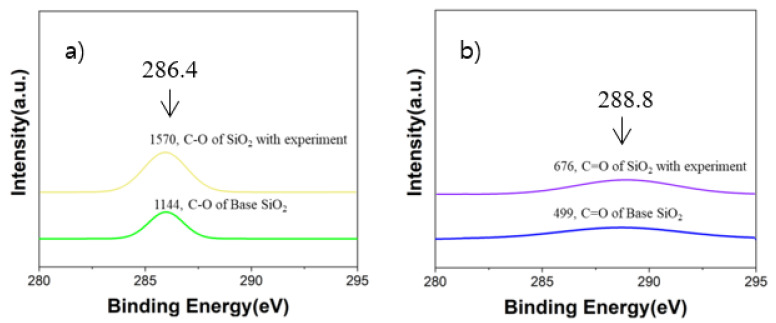
Binding energy peak of the C-O bonding on the base SiO_2_ and after the vacuum carbon vaporization experiment (**a**); Binding energy peak of the C=O bonding on the base SiO_2_ and after the vacuum carbon vaporization experiment (**b**).

Figure 8 shows the XPS results of analyzing the binding energy of carbon and carbon compounds on the base SiO_2_ wafer before and after the vacuum carbon vaporization experiment. More specific XPS results regarding the binding energy of C–O/C=O bonding are shown in Figure 9. During the XPS analysis, 284.8 eV was used as a reference for the binding energy of the C-C bonding, which has commonly been used in many previous studies [25]. Based on the reference bonding energy, 286.4 eV and 288.8 eV were applied as the binding energies of the C-O bonding and C=O bonding, respectively [25]. Through the vacuum carbon vaporization experiment, it was observed that the intensities of the C-O bonding and C=O bonding on the SiO_2_ wafer increased. It is considered that the carbon powder reacted with the remaining oxygen in the chamber to form CO and CO_2_ gases and that it was then adsorbed on the SiO_2_ wafer surface. Although the chamber was maintained in a vacuum state, the degree of 10^−3^ torr was in the low-vacuum region, and the vacuum was not sufficient to prevent all the reactions with oxygen. In this case, it can be assumed that there is a possibility that oxygen molecules remained and reacted with the carbon sources in the chamber.

**Figure 10:**
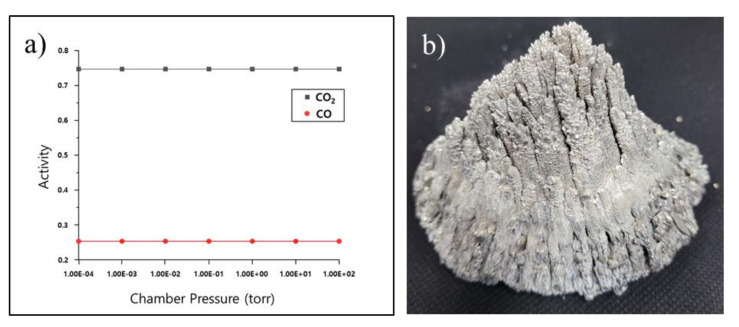
Activities of CO_2_ and CO according to the pressure changes in the chamber at 600 °C (**a**) and Mg crown (**b**).

Figure 10a shows the activities of CO_2_ and CO according to the pressure change in the chamber at 600 °C. These activities were calculated using the Factsage 8.1&FactPS database. It was assumed that 1 mol of carbon and 1 mol of air were in the chamber. The partial pressure of O_2_ in 1 mol of air was changed. The temperature of the chamber was fixed at 600 °C, and the activities of CO_2_ and CO according to the change in the vacuum degree in the chamber were calculated. From the calculation, a small amount of carbon reacted with O_2_ to generate a gas phase, and the activities of CO_2_ and CO in the gas phase were constant at 0.75 and 0.25, respectively, regardless of the vacuum degree. The vacuum carbon vaporization experiment was conducted under conditions similar to those of the vacuum distillation process used to produce high-purity pure Mg. It is possible that CO and CO_2_ could have been generated and adsorbed on the Mg crown during the production process of the high-purity pure magnesium. The very porous Mg crown with a very large surface area, as shown in Figure 10b, provides a favorable environment for CO and CO_2_ to be adsorbed on its surface. From these results, it can be suggested that high-purity Mg crowns contain a higher level of carbon sources such as CO and CO_2_ than commercial-purity Mg crowns. Subramanian et al. [26] conducted a study on the improvement of mechanical properties in Mg-Al alloys by introducing CO_2_ into the melt. They reported that, due to the interaction between carbon dioxide and aluminum, Al_4_C_3_ particles formed in the melt at 750 °C. The in situ-formed Al_4_C_3_ particles served as nucleation sites for α-Mg grains, resulting in grain refinement and enhancement of the mechanical properties of Mg-Al alloys. Additionally, Yan Liu et al. [27] reported that the addition of gaseous carbon dioxide (CO_2_) led to grain refinement in Mg-8 wt.% Al alloys. They found that the grain refinement efficiency was primarily attributed to CO_2_ gas, which facilitated the formation of Al_4_C_3_ in the melt at 740 °C. The Al_4_C_3_ particles were reported to act as the dominant heterogeneous nucleation substrate for α-Mg grains. Therefore, it can be suggested that the finer grain size in high-purity AZ91 alloy compared to commercial-purity AZ91 alloy can be attributed to the higher amount of carbon sources, which promote the in situ formation of Al-C particles in the high-purity AZ91 alloy than in the commercial-purity AZ91 alloy. Additionally, it is believed that these carbon sources are introduced into the high-purity AZ91 alloy through the vacuum distillation process for producing high-purity pure Mg ingots used in the manufacturing of high-purity AZ91 alloys.

## 4. Conclusions

In this study, the factors for the finer grain structure of high-purity AZ91 alloys compared to commercial-purity AZ91 alloys were investigated. The results showed that the higher carbon content in high-purity AZ91 alloys was a key factor in the formation of powerful nucleants that facilitated grain refinement. SEM analysis of rapidly solidified high-purity AZ91 alloy ribbon samples confirmed the presence of Al-C particles, which are known to act as nucleants for α-Mg. Vacuum distillation experiments using a SiO_2_ wafer as a substrate revealed the presence of more carbon sources on the top surface of the wafer, indicating that more carbon sources were likely transferred to the high-purity Mg crowns and ingots during the manufacturing process. These carbon sources reacted with the Al in the Mg melts, forming Al-C compounds that acted as effective nucleants for α-Mg. Overall, this study presents a new perspective on the mechanisms of grain refinement in high-purity AZ91 alloys and suggests new possibilities.

## Figures and Tables

**Table 1:** Chemical compositions of the commercial-purity and high-purity AZ91 alloys.

Alloy	Al	Zn	Mn	Si	Fe	Cu	Ni	Mg
Commercial Purity AZ91	8.93	0.57	0.250	0.015	0.0022	0.0016	0.0012	Bal.
High Purity AZ91	8.97	0.69	0.006	0.0015	0.0010	0.0006	0.0012	Bal.

## Data Availability

The data used to support the findings of this study are available from the corresponding author upon request.

## References

[1] Song J., Chen J., Xiong X., Peng X., Chen D., Pan F. (2022). Research Advances of Magnesium and Magnesium Alloys Worldwide in 2021. J. Magnes. Alloy..

[2] Kaya A.A. (2020). A Review on Developments in Magnesium Alloys. Front. Mater..

[3] Mordike B.L., Ebert T. (2001). Magnesium Properties—Applications—Potential. Mater. Sci. Eng. A.

[4] Easton M.A., Qian M., StJohn D.H. (2011). Grain Refinement in Alloys: Novel Approaches. Encyclopedia of Materials: Science and Technology.

[5] Qian M., StJohn D.H., Frost M.T. (2002). Characteristic Zirconium-Rich Coring Structures in Mg-Zr Alloys. Scr. Mater..

[6] Lee Y.C., Dahle A.K., Stjohn D.H. (2000). The Role of Solute in Grain Refinement of Magnesium. Metall. Mater. Trans. A Phys. Metall. Mater. Sci..

[7] Jung S.S., Son Y.G., Park Y.H., Lee Y.C. (2022). A Study on the Grain Refining Mechanisms and Melt Superheat Treatment of Aluminum-Bearing Mg Alloys. Metals.

[8] Stjohn D.H., Easton M.A., Qian M., Taylor J.A. (2013). Grain Refinement of Magnesium Alloys: A Review of Recent Research, Theoretical Developments, and Their Application. Metall. Mater. Trans. A Phys. Metall. Mater. Sci..

[9] Tamura Y., Haitani T., Yano E., Motegi T., Kono N., Sato E. (2002). Grain Refinement of High-Purity Mg-Al Alloy Ingots and Influences of Minor Amounts of Iron and Manganese on Cast Grain Size. Mater. Trans..

[10] Tamura Y., Yagi J., Haitani T., Motegi T., Kono N., Tamehiro H., Saito H. (2003). Observation of Manganese-Bearing Particles in Molten AZ91 Magnesium Alloy by Rapid Solidification. Mater. Trans..

[11] Cao P., Qian M., StJohn D.H. (2004). Effect of Iron on Grain Refinement of High-Purity Mg-Al Alloys. Scr. Mater..

[12] Cao P., Qian M., Stjohn D.H. (2005). Native Grain Refinement of Magnesium Alloys. Scr. Mater..

[13] Srinivasan A., Pillai U.T.S., Pai B.C. (2005). Microstructure and Mechanical Properties of Si and Sb Added AZ91 Magnesium Alloy. Metall. Mater. Trans. A.

[14] Han M., Zhu X., Gao T., Liu X. (2017). Revealing the Roles of Al4C3and Al8Mn5during α-Mg Nucleation in Mg-Al Based Alloys. J. Alloys Compd..

[15] Tamura Y., Haitani T., Kono N. (2006). Liquid Solubility of Manganese and Its Influence on Grain Size of Mg-Al Alloys. Mater. Trans..

[16] Men H., Jiang B., Fan Z. (2010). Mechanisms of Grain Refinement by Intensive Shearing of AZ91 Alloy Melt. Acta Mater..

[17] Wang X.J., Chen X.D., Xia T.D., Yu W.Y., Wang X.L. (2004). Influencing Factors and Estimation of the Cooling Rate within an Amorphous Ribbon. Intermetallics.

[18] Neelameggham N.R. (2013). Primary Production of Magnesium.

[19] Hwang D.J., Yu Y.H., Lee J.D. (2021). A Study on the Characteristics of Manufactured Mg Crown on the Calcining Conditions of Dolomite. Korean Chem. Eng. Res..

[20] Baek U.H., Lee B.D., Lee K.W., Han G.S., Han J.W. (2016). Study of the Thermal Reduction Behavior of Dolomite by the Pidgeon Process. J. Korean Inst. Met. Mater..

[21] Revel G., Pastol J.L., Rouchaud J.C., Fromageau R. (1978). Purification of Magnesium by Vacuum Distillation. Metall. Trans. B.

[22] Tian Y., Zhang X., Qu T., Lyu F., Du H., Shi L., Yang B., Dai Y. (2021). Technical Research on Vacuum Distillation to Purify Magnesium to 99.99% Purity. Mater. Res. Express.

[23] Wang Y.C., Tian Y., Qu T., Yang B., Dai Y.N., Sun Y.P. (2014). Purification of Magnesium by Vacuum Distillation and Its Analysis. Mater. Sci. Forum.

[24] Tamura Y., Haitani T., Kono N., Motegi T., Sato E. (1998). Vacuum Distillation of Magnesium. Keikinzoku/J. Jpn. Inst. Light Met..

[25] Fang D., He F., Xie J., Xue L. (2020). Calibration of Binding Energy Positions with C1s for XPS Results. J. Wuhan Univ. Technol. Mater. Sci. Ed..

[26] Subramanian J., Guan K.C., Kuma J., Gupta M. (2011). Feasibility Study on Utilizing Carbon Dioxide during the Processing of Mg-Al Alloys. J. Mater. Process. Technol..

[27] Liu Y., You G., Gao F., Long S., Liu Q., Shao J., Ao S., Li X. (2017). Effect of Gaseous Carbon Dioxide on Grain Refinement in Mg-8Al Alloy. Mater. Sci. Technol..

